# Impact of polysialylated CD56 on natural killer cell cytotoxicity

**DOI:** 10.1186/1471-2172-8-13

**Published:** 2007-08-06

**Authors:** Jeannette M Moebius, Darius Widera, Juergen Schmitz, Christian Kaltschmidt, Christoph Piechaczek

**Affiliations:** 1 Miltenyi Biotec GmbH, Bergisch Gladbach, Germany; 2 University of Witten-Herdecke, Witten, Germany

## Abstract

**Background:**

Siglec-7, a sialic acid binding inhibitory receptor expressed by NK cells is masked *in vivo *by a so far unknown ligand. It shows a strong binding prevalence for α-2,8-linked disialic acids *in vitro*.

**Results:**

Here we describe the expression of PSA-NCAM (α-2,8-linked polysialic acid modified NCAM) on functional adult peripheral blood natural killer cells and examine its possible role in masking Siglec-7. Unmasking of Siglec-7 using *Clostridium perfringens *neuraminidase massively reduces NK cell cytotoxicity. By contrast a specific removal of PSA using Endo-NF does not lead to a reduction of NK cell cytotoxicity.

**Conclusion:**

The results presented here therefore indicate that PSA-NCAM is not involved in masking Siglec-7.

## Background

Siglec-7 (p75/AIRM1) is a sialic acid binding inhibitory receptor expressed on NK and NKT cells and to a lesser extend also on monocytes [1]. It shows a prevalence for binding α-2,8-linked or branched α-2,6-linked sialic acids *in vitro *and is constitutively masked by a endogenous ligand *in vivo *[2] which has not been identified yet. It has been shown that trans-activation of unmasked Siglec-7 leads to an inhibition of NK cell cytotoxicity [2,3]. Natural killer cells are characterized by the expression of CD56 (neural cell adhesion molecule, NCAM). NCAM is an immunoglobulin-like cell adhesion molecule (IgCAM) and was the first vertebrate protein demonstrated to be glycosylated with polysialic acid (PSA), which is a homomeric polymer of α-2,8-sialic acid. Until now only very few proteins have been reported to be modified with PSA [4,5].

Our question was whether CD56 on NK cells is polysialylated and if PSA-NCAM could be the endogenous ligand masking Siglec-7. For this purpose we performed a FACS analysis examining PSA expression on peripheral blood. The impact of PSA on NK cell cytotoxicity was determined by killing assays after specific enzymatic removal of sialic acids.

## Results

### Detection of PSA expression in human peripheral blood

**Figure 1:**
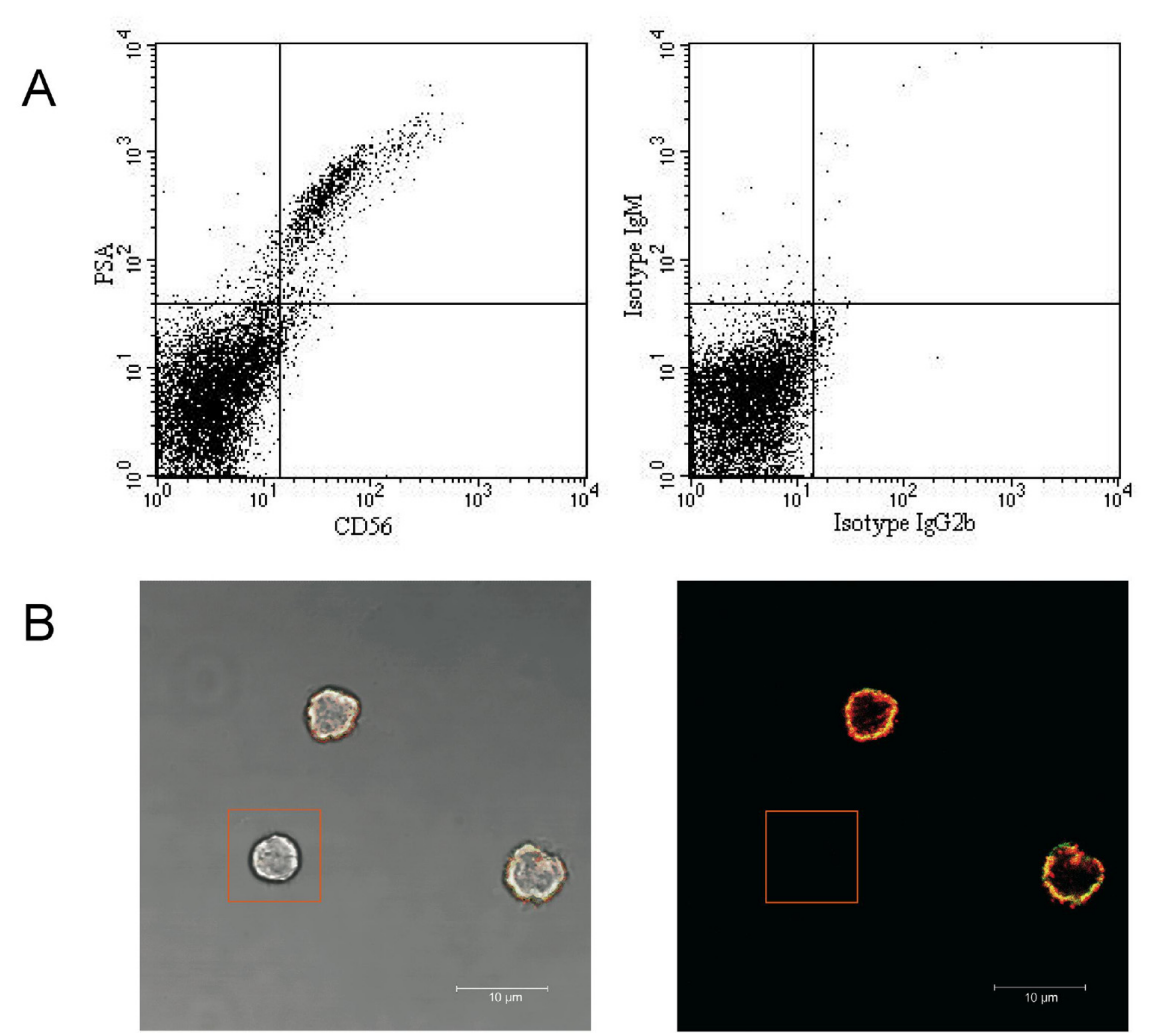
**NK cells express PSA-NCAM**. (A) FACS analysis of co-expression of PSA and CD56 in peripheral blood mononuclear cells shows a strong correlation of staining intensities. Data shown are representative of several independent experiments. (B) Expression of PSA (red) and CD56 (green) by NK cells examined via confocal microscopy. Rectangle indicates an unlabeled PBMC cell.

To detect PSA^+ ^cells in peripheral blood we used an antibody which binds PSA of at least twelve α-2,8-linked sialic acid residues [6,7]. Co-staining of PSA and CD56 not only shows co-expression but also a strong correlation of staining intensity, which indicates that CD56 on NK cells, is polysialylated (Fig. 1A). Co-expression of PSA and CD56 was also verified by immunocytochemical staining (Fig. 1B). Polysialylation of NCAM on NK cells has also been suggested by Lanier et al. who sowed excessive sialylation of NCAM, but the nature of the linking of sialic acid monomers was not investigated [8].

**Figure 2:**
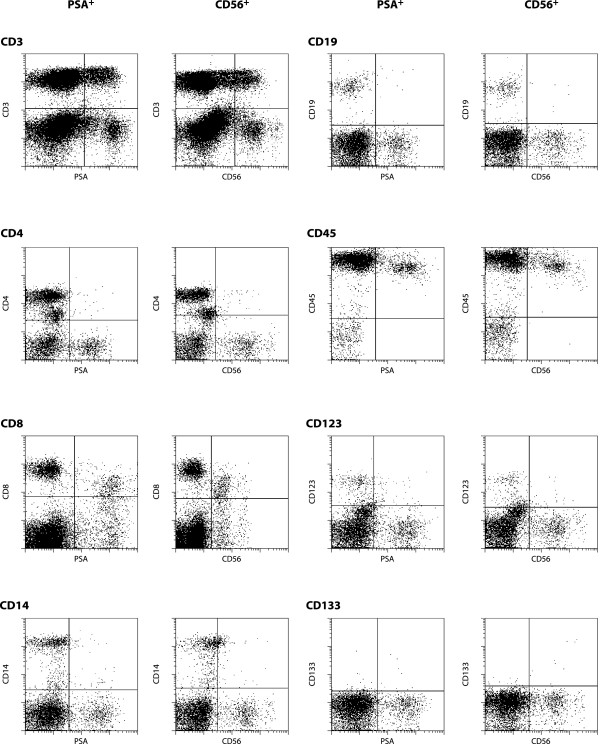
**Expression of PSA and CD56 in peripheral blood**. FACS analysis of PSA and CD56 expression in PBMC via co-staining with CD3, CD4, CD8, CD14, CD19, CD45, CD56, CD123 and CD133.

To exclude PSA expression by other hematopoietic cell lineages we compared expression of CD56 with anti PSA staining. For this purpose co-stainings with several lineage markers for T cells (CD3, CD4, CD8), monocytes (CD14), B cells (CD19), NK cells (CD56), dendritic cells and basophiles (CD123) and stem cells (CD133) were performed. FACS analysis showed PSA staining on CD56^+^cells and on small subpopulations of CD3^+ ^and CD8^+ ^cells, which also express CD56 (Fig. 2). Our data indicates that PSA-NCAM is expressed by NK and NKT cells, cell types which are also known to express siglec-7 [1].

### Characterization of polysialyltransferase expression in NK cells

**Figure 3:**
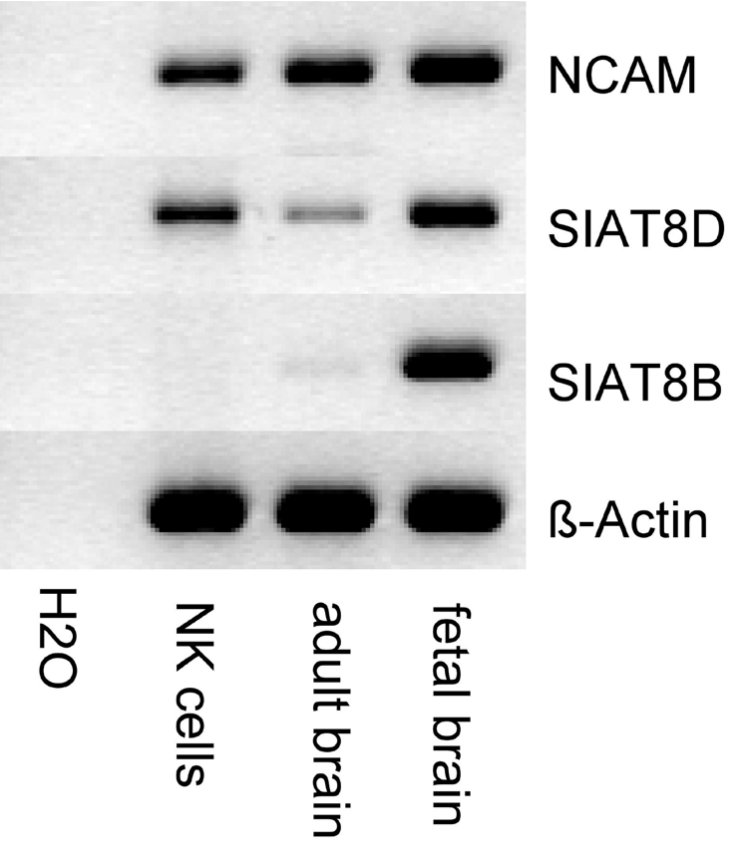
**Analysis of polysialyltransferase expression**. PCR analysis of cDNA derived from CD56^+ ^NK cells and human fetal and adult brain regarding their expression of NCAM and the two polysialyltransferases SIAT8D and SIAT8B. cDNA concentration was normalized via the β-actin housekeeping gene.

Two enzymes have been shown to be independently able to synthesize PSA, the polysialyltransferases SIAT8D (ST8SiaIV, PST) and SIAT8B (ST8SiaII, STX). To determine which polysialylating enzymes are involved in the expression of PSA on NK cells reverse transcriptase PCR was performed. We compared SIAT8D and SIAT8B mRNA expression in adult CD56^+ ^NK cells and in human adult and fetal brain. Expression of SIAT8D was found in NK cells as well as in both brain samples. In contrast SIAT8B was expressed predominantly in fetal brain, but the expression was drastically reduced in adult brain and could not be detected in adult NK cells (Fig. 3). These findings suggest that only SIAT8D contributes to NCAM polysialylation in peripheral blood NK cells. SIAT8D expression by NK cells was also reported recently by Avril and colleagues [9].

### Functionality of NK cells after separation via PSA

**Figure 4:**
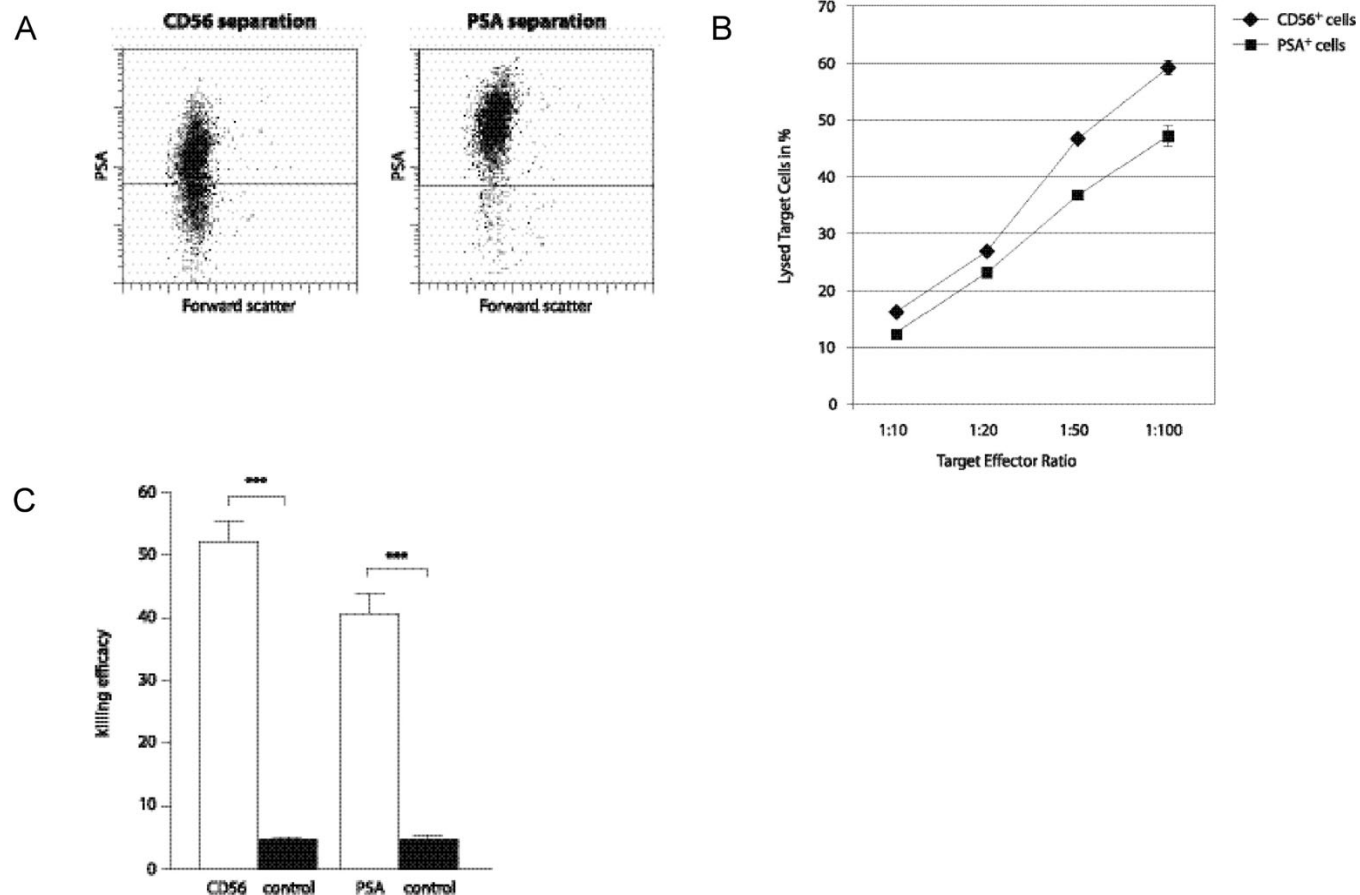
**PSA^+ ^cells are functional NK cells**. (A) PSA expression on cells isolated via PSA or CD56. (B) NK cells isolated either via CD56 or PSA were assessed for their ability to lyse K562 target cells in a 4 h-killing assay. Data shown are the average mean ± SEM from one out of three independent experiments. (C) Comparison of killing efficiencies at the effector target ratio 50:1. Data shown are the average mean ± SEM from three independent experiments (p < 0.001). Control refers to the vitality of the K562 cell line alone.

In order to provide functional evidence, PSA^+ ^cells and via CD56 isolated NK cells were compared using K562 killing assays. As shown in Fig. 4A, cells separated either via CD56 or PSA show expression of PSA. Separated cells were co-cultured with the target cell line K562 for four hours followed by a killing efficiency determination. A comparative killing assay showed only a small difference in killing efficiency (Fig. 4B). Comparison of killing efficiencies at the effector target ratio 50:1 showed no significant reduction of killing ability by the separation strategy (Fig. 4C). The slightly reduced killing efficiency of cells separated via PSA could be caused by the large labeling complex (anti PSA + anti IgM.PE + anti PE microbeads) due to steric inhibition. These data indicate that cells separated via PSA are functional NK cells.

### Impact of PSA expression on NK cell function

**Figure 5:**
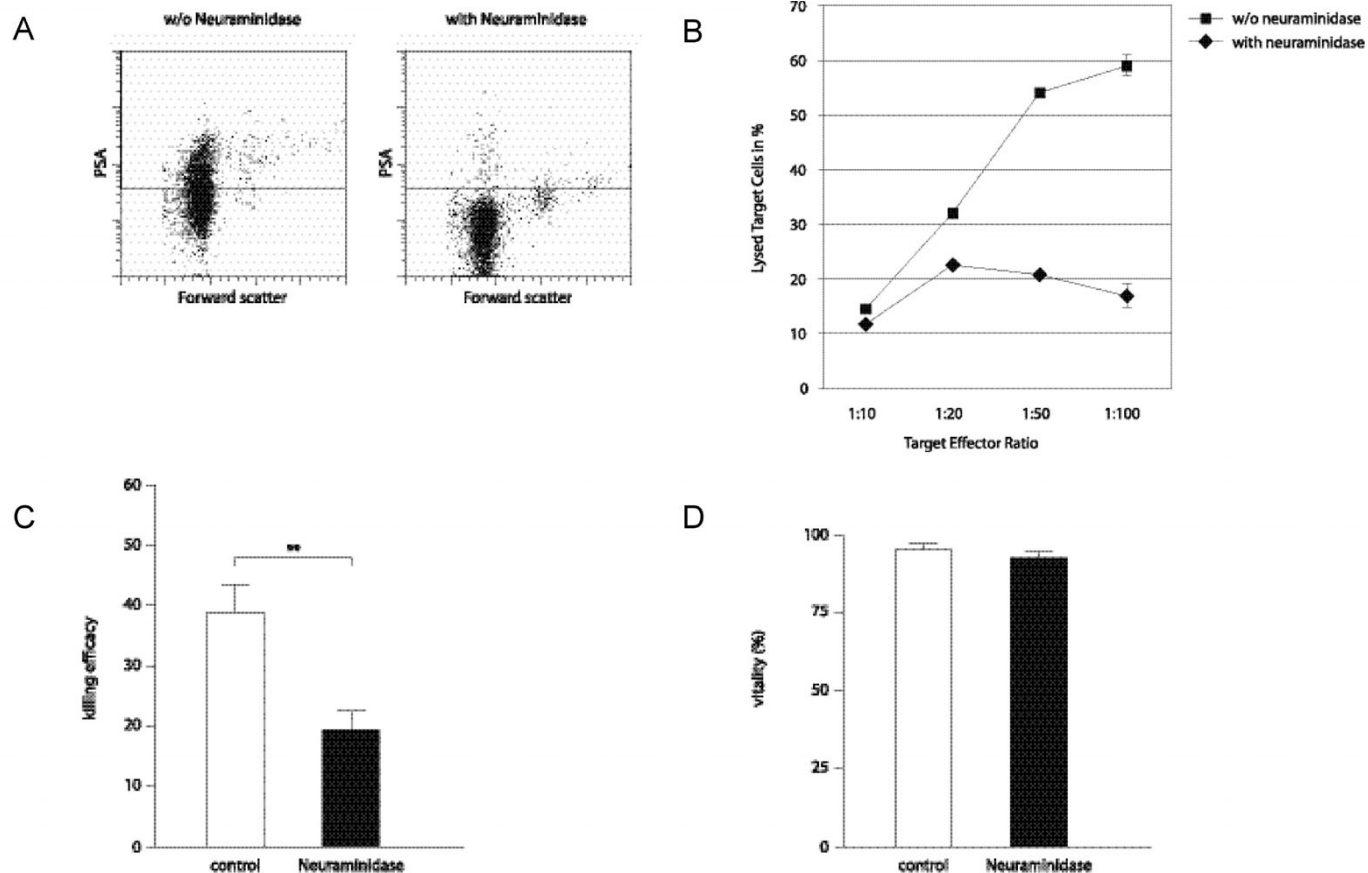
**Neuraminidase treatment reduces NK cell cytotoxicity**. (A) FACS analysis of PSA expression with and without *Clostridium perfringens *Neuraminidase treatment. (B) CD56^+ ^cells with and without Neuraminidase treatment were investigated in a 4 h-killing assay. Data shown are the average mean ± SEM from one out of three independent experiments (C) Comparison of killing efficiencies at the target effector ratio 1:50 shows a significant reduction of cytotoxicity after Neuraminidase treatment. Data shown are the average mean ± SEM from three independent experiments (p = 0.0025). Control refers to the killing efficiency of untreated NK cells. (D) Comparison of vitality with and without neuraminidase treatment shows no significant difference. Data shown are the average mean ± SEM from three independent experiments.

**Figure 6:**
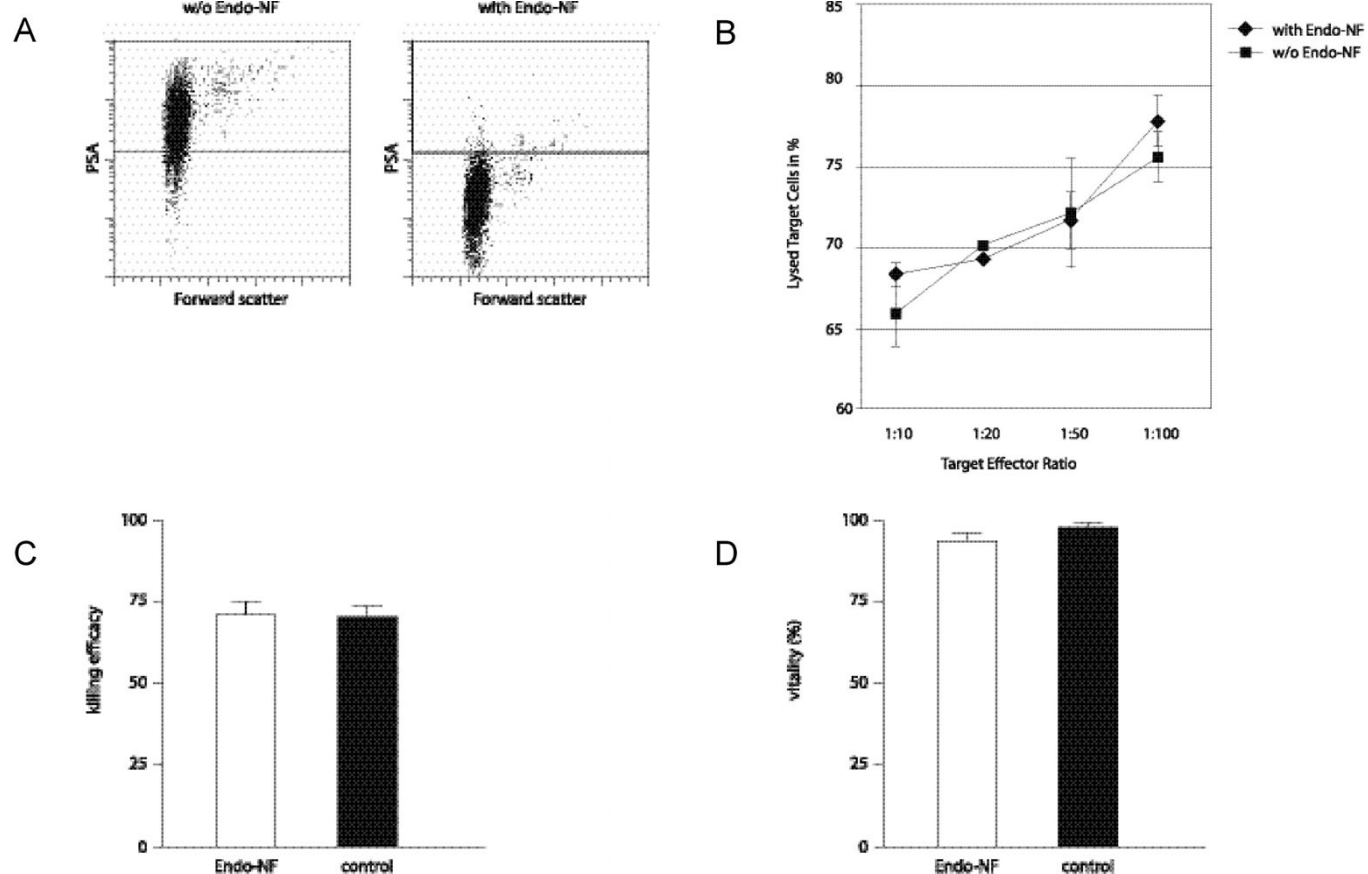
**Endo-NF treatment has no impact on NK cell cytotoxicity**. (A) FACS analysis of PSA expression with and without Endo-NF treatment. (B) NK cells separated via CD56 were investigated in a 4 h-killing assay with and without Endo-NF treatment. Data shown are the average mean ± SEM from one out of three independent experiments. (C) Comparison of killing efficiencies at the target effector ratio 1:50 shows no difference of treated and untreated cells. Data shown are the average mean ± SEM from three independent experiments. Control refers to the killing efficiency of untreated NK cells. (D) Comparison of vitality with and without Endo-NF treatment shows no significant loss of vitality. Data shown are the average mean ± SEM from three independent experiments.

To investigate the potential impact of polysialic acid on the killing ability of NK cells, sialic acid residues were removed enzymatically with *Clostridium perfringens *neuraminidase or Endo-NF and NK cells were again tested in a killing assay. *Clostridium perfringens *neuraminidase is able to remove all sialic acid residues (α-2,3, α-2,6 and α-2,8-linked sialic acids) from the cell surface while Endo-NF removes only α-2,8-linked sialic acid residues. After enzymatic treatment the vitality was not reduced significantly (91% after neuraminidase treatment in comparison to 96.7% without neuraminidase treatment, Fig. 5D; and 93,5% after Endo-NF treatment and 97,5% without treatment, Fig. 6D). As can be seen in Fig 5A and 6A only few PSA expressing cells are remaining after neuraminidase or Endo-NF treatment. Only 5% of neuraminidase treated cells and 2% of Endo-NF treated cells show re-expression of PSA on the cell surface after 4 h culture in RPMI/2 mM L-Glutamine/10% FCS (data not shown) indicating that PSA is only weakly re-expressed during the killing assay. The comparative killing assay revealed a strongly reduced killing efficiency of neuraminidase treated NK cells (Fig. 5B). Comparison of killing efficiencies at the target effector ratio 1:50 showed a highly significant reduction of killing ability of NK cells after neuraminidase treatment (Fig. 5C) while Endo-NF treatment had no impact on the killing efficiency (Fig 6B/C).

## Discussion

The role of PSA-NCAM in the developing brain is well characterized, whereas only little is known about PSA-NCAM expression and function in other organs.

The results presented here show PSA-NCAM expression on functional human peripheral blood NK cells, which can be isolated via PSA without loss of function.

Phenotyping experiments with several lineage markers for T cells (CD3, CD4, CD8), monocytes (CD14), B cells (CD19), NK cells (CD56), dendritic cells and basophiles (CD123) and stem cells (CD133) demonstrated expression of PSA on CD56^+^/CD45^+ ^NK cells and on CD3^+^/CD8^+^/CD45^+^/CD56^+ ^NKT cells.

Functionality of PSA^+ ^cells was analyzed using killing assays, showing that NK cells separated according to their expression of PSA showed only a slightly reduced killing capacity compared to NK cells separated using CD56 microbeads. The large labeling complex used to isolate PSA^+ ^cells could cause this reduction due to steric inhibition. PSA expression has also been described on decidual NK cells (dNK cells) [10] which are located in the human placenta where they play an important role in maintaining pregnancy. In contrast to peripheral blood derived NK cells dNK cells show only week cytotoxicity. And as their expression pattern differs from peripheral blood NK cells, even a different NK cell lineage arising from a different progenitor was suggested [11].

PCR analysis of the expression of the two polysialyltransferases SIAT8D and SIAT8B showed that NK cells only express SIAT8D. It has been shown that SIAT8D plays a predominant role in NCAM polysialylation and that mRNA levels strongly correlate with protein expression [12]. We could show that SIAT8D is strongly expressed on mRNA level in NK cells, which suggests that SIAT8D is the main polysialylating enzyme in adult NK cells. Its expression also provides additional evidence for polysialylation of NCAM.

As has been described before, enzymatic removal of all sialic acid residues with *Clostridium perfringens *Neuraminidase reduced the killing efficiency of CD56^+ ^cells dramatically due to de-masking of siglec-7 [2]. However Endo-NF treatment of NK cells did not lead to a reduction of killing efficiency. A reduction of PSA polymerization degree is supposed to have a major effect on PSA function. We thus conclude that PSA is not involved in masking Siglec-7.

Removal of PSA using Endo-NF creates a rest of four α-2,8-linked sialic acid residues [13]. The PSA antibody (Men-B) used in the experiments above recognizes PSA of a minimum chain length of twelve sialic acid residues [14]. We thus conclude that after Endo-NF treatment a minimum of four and a maximum of eleven α-2,8-linked sialic acid residues are still left on the NK cell surface. The fact that removal of sialic acid residues with Endo-NF does not lead to a reduced cytotoxicity of NK cells gives rise to the speculation that the unknown endogenous ligand of Siglec-7 consists either of maximal four α-2,8-linked sialic acid residues or α-2,6-linked branched sialic acid residues. Other candidates for the masking ligand of Siglec-7 have already been identified. Avril et al. for example showed recently expression of a so far uncharacterized α-2,8-disialylated O-linked glycan by NK cells [9].

The role of PSA-NCAM on NK cells remains unknown. PSA-NCAM has been characterized in the nervous system, where it has an impact on cell migration, axonal path finding, neurite outgrowth, fasciculation of neurites and also modulates AMPA receptors [15] (for NCAM and PSA-NCAM interactions and signal transduction see [16] for review). Muscle cells, including cardiac myocytes also express PSA-NCAM during development. Furthermore PSA can directly modulate the homophilic binding potential of NCAM [17] and also interactions with other proteins. And Agrin, a heparan sulfate proteoglycan of the extracellular matrix has been suggested to bind the heparin-binding site of NCAM and PSA [18]. Signaling of brain-derived neurotrophic factor (BDNF) [19] and platelet derived growth factor (PDGF) [20] is affected by the PSA moieties bound to NCAM.

## Conclusion

In conclusion we could show expression of PSA-NCAM on functional human peripheral blood NK cells. Our data strongly indicates that the polysialyltransferase SIAT8D is responsible for PSA expression in NK cells. Killing assays after specific removal of PSA revealed that elimination of PSA has no effect on NK cell cytotoxicity and thus indicate that polysialic acid is not the endogenous ligand masking Siglec-7.

## Methods

### Cell culture

K562 cell line was cultured in RPMI (Miltenyi Biotec GmbH, Bergisch Gladbach, Germany), 10% FCS (PAA, Brisbane, Australia), 2 mM L-Glutamine (Gibco, Paisley, Scotland, UK) and 1% Penicillin-Streptomycin (10,000 U/ml, Gibco) at 37°C, 5% CO_2_. Medium was changed twice weekly.

### Flow cytometric analysis

For flow cytometric analysis Fc receptors were blocked with FCR Blocking Reagent (Miltenyi Biotec GmbH) and incubated for 10 minutes at 4–8°C with an anti PSA monoclonal antibody (clone MenB, 2 μg/ml). After washing once with PBS/2 mM EDTA cells were incubated with a phycoerythrin (PE) conjugated anti IgM monoclonal antibody (clone X-54) or a PE conjugated anti CD56 monoclonal antibody (clone AF12-7H3, all Miltenyi Biotec GmbH) and the following antibodies: FITC (fluorescein isothiocyanate) conjugated anti CD4 (clone M-T321), anti CD14 (clone TÜK4), anti CD123 (clone AC145), allophycocyanin (APC) conjugated anti CD8 (clone BW135/80), anti CD16 (clone VEP13), anti CD19 (clone LT19), anti CD45 (clone 5B1), anti CD133 (clone 293C3), anti CD335 (clone 9E2) (all Miltenyi Biotec GmbH). For CD3 co-staining an anti CD3-APC (clone SK7, BD Bioscience, San Diego, CA, USA) was used. FACS analysis after cell separation was done using a FITC conjugated anti CD56 (clone NCAM16.2, BD Pharmingen, BD Bioscience, San Diego, CA, USA). Isotype controls were purchased from Pharmingen, BD Bioscience.

FACS analysis was performed using a FACS Calibur flow cytometer (Becton Dickinson, Heidelberg, Germany). Dead cells were excluded via propidium iodide staining. Data analysis was performed with BD CELLQuest software.

### Immunocytochemistry

Untouched NK cells were isolated from peripheral blood mononuclear cells (PBMC) using the NK Cell Isolation Kit II (Miltenyi Biotec GmbH) according to manufacturer's instructions.

In order to show specificity of immunocytochemical staining 1 × 10^6 ^PBMC were added to 1 × 10^6 ^cells of the positive fraction and the cells were fixed with 3.7% neutral buffered formalin for 10 minutes at room temperature. After washing twice, unspecific binding sites were blocked by incubation with PBS/5% normal goat serum (Vector Laboratories, Burlingame, CA, USA) and cells were incubated with anti PSA (Clone MenB, 2 μg/ml) and anti CD56 (clone AF12-3H7, 5 μg/ml, Miltenyi Biotec GmbH) for 1 hour at 4–8°C. After washing twice with PBS/5% normal goat serum, cells were incubated with rat anti murine IgG1-Alexa488 (clone CLX56, labeled using the Alexa Fluor 488 Protein Labelling Kit, Molecular Probes, Invitrogen, Karlsruhe, Germany) and goat anti rat IgM-RhodamineX (clone C48-6, 1:500, Pharmingen, BD Bioscience) for 1 hour at 4–8°C. After washing with PBS/5% normal goat serum, 2 × 10^5 ^cells were spun onto microscope slides using a Shandon Cytospin 3 Cytocentrifuge (Thermo Electron Corp., Waltham, MA, USA) at 800 rpm for 5 minutes, mounted with Fluoromount G (Southern Biotech, Birmingham, USA) and analysed with a Leica DMIRE2 confocal microscope (Leica Microsystems, Wetzlar, Germany) using Leica Confocal Software.

### Isolation of CD 56^+ ^and PSA^+ ^cells

CD56^+ ^and PSA^+ ^cells were separated from PBMC using the MACS technology (Miltenyi Biotec GmbH). Briefly, cells were incubated with magnetic bead conjugated CD56 monoclonal antibody (clone AF12-7H3, Miltenyi Biotec GmbH) and separated over one MS column. The purity ranged from 91.3% to 98.3% (average 95.7%) and was checked by FACS analysis using anti PSA (clone MenB), anti IgM-PE (clone X54) and anti CD56.FITC (clone NCAM16.2).

PSA^+ ^cells were separated using an anti PSA monoclonal antibody (clone MenB). Subsequently, cells were incubated with a PE conjugated anti IgM secondary antibody (clone X54) and magnetic labeling was performed using anti PE microbeads (Miltenyi Biotec GmbH). Cells were separated over two MS columns and the purity ranged from 91.4% to 96.1% (average 94.5%).

### K562 killing assay

The killing efficiency of CD56^+ ^cells and PSA^+ ^cells was determined via the killing of the MHC I&II negative target cell line K562.

K562 cells were labeled with CFDA-SE (carbofluorescein diacetate succinimidylester, 2.5 ng/ml, Molecular Probes) in medium without FCS. After two minutes the labeling reaction was stopped by adding FCS, followed by a washing step. Then the cells were incubated in cell culture medium until needed.

The target cells were incubated with CD56^+ ^or PSA^+ ^cells in the target effector ratios 1:10, 1:20, 1:50 and 1:100 in complete medium at 37°C. After 4 h the killing reaction was stopped by putting the cells on ice. The cells were analyzed by FACS analysis and dead cells were discriminated using propidium iodide. Data analysis was performed by BD CELLQuest software. The killing efficiency was determined by the ratio of the number of dead CFDA-SE^+ ^cells to the total number of CFDA-SE^+ ^cells.

### Reverse transcriptase PCR

Total RNA of 1 × 10^6 ^CD56^+ ^cells was extracted using the NucleoSpin RNA II Kit (Macherey & Nagel, Dueren, Germany) according to the manufacturer's instructions. cDNA was synthesized in a 30 μl reaction mixture containing 300 U SuperscriptII, 1× first strand buffer (0.3 μM, Invitrogen), dNTP (200 μM each, PEQLAB, Erlangen, Germany), Oligo(dT)18 (5 μg) and (N6) random hexamere (0.2 μg, both Metabion, Martinsried, Germany).

Human normal adult brain cDNA (1 μl per PCR reaction) and human fetal brain cDNA (2 μl per PCR reaction) were purchased from Biochain Institute, Inc (Hayward, CA, USA).

PCR was performed in a 50 μl reaction mixture containing 1.25 units Taq polymerase, 1× reaction buffer, 2 mM MgCl_2 _(Fermentas International Inc., Burlington, Ontario, Canada), 200 μM of each dNTP (PEQLAB, Erlangen, Germany) and 200 nM primers (Metabion). cDNA concentrations were normalized via the β-actin housekeeping gene. The following primers were used: SIAT8B (forward: agacctggtaaccatgaaccc, reverse: tgggaggtgtagccatacttg), SIAT8D (forward: ccaatgaagaatcgcaggtt, reverse: cttagggaagggccagaatc), β-actin (forward: gagaagatgacccagatcatgt, reverse: catctcttgctcgaagtccag).

The cycling conditions comprised an initial denaturation of 5 min at 94°C and 30 cycles of 1 min at 94°C, 1 min at 60°C and 1 min at 72°C followed by a final elongation of 10 min at 72°C.

### Enzymatic treatment

2 × 10^6 ^CD56^+ ^cells/ml were cultured in RPMI/10% FCS/2 mM L-glutamine containing 0.125 U/ml *Clostridium perfringens *Neuraminidase type V (Sigma Chemical Co., Deisendorf, Germany) or 1.5 ng/ml Endo-NF derived from NF bacteriophage (kind gift from M. Mühlenhoff, Medizinische Hochschule Hannover, Hannover, Germany) for 12 h to 16 h. Untreated CD56^+ ^cells were cultured in RPMI/10% FCS/2 mM L-glutamine at 37°C, 5% CO_2_.

### Statistics

Statistical significance of the killing efficiency of NK cells isolated via their CD56 or PSA expression was determined using two-way ANOVA followed by post hoc t-test with Bonferroni correction. Statistical significances of the killing efficiency and the vitality of CD56^+ ^NK cells with and without enzymatic treatment were determined by unpaired t-test. Differences between two conditions at p < 0.05 were considered as statistically significant.

## Authors' contributions

JM carried out the FACS analysis, immunocytochemical staining, PCR analysis, killing assays and drafted the manuscript. DW carried out the statistical analysis and helped to draft the manuscript. JS, CK and CP participated in the study design and helped to draft the manuscript. All authors read and approved the final manuscript.
